# Selection of Treatment Regimens for Recurrent Cervical Cancer

**DOI:** 10.3389/fonc.2021.618485

**Published:** 2021-02-02

**Authors:** Xiaopei Chao, Xiaochen Song, Huanwen Wu, Yan You, Ming Wu, Lei Li

**Affiliations:** ^1^ Department of Obstetrics and Gynecology, Peking Union Medical College Hospital, Beijing, China; ^2^ Department of Pathology, Peking Union Medical College Hospital, Beijing, China

**Keywords:** cervical cancer, recurrence, radiotherapy, chemotherapy, surgery, progression-free survival

## Abstract

**Objective:**

The selection of individualized treatment for recurrent cervical cancer is challenging. This study aimed to investigate the impact of various therapies on survival outcomes after recurrence.

**Methods:**

Eligible patients were diagnosed with recurrent cervical cancer between March 2012 and April 2018. Postrecurrence progression-free survival (PFS) and overall survival (OS) were investigated in the whole cohort and in subgroups, categorized by recurrence site and prior radiotherapy history, using a multivariate model that incorporated treatment for primary and recurrent tumors, histological pathology, and FIGO staging.

**Results:**

Two hundred and sixty recurrent cervical cancer patients were included. As of March 1, 2020, the median postrecurrence PFS and OS were 7.0 (range 0-94) and 24.0 (1.8-149.1) months, respectively. In a multivariate model measured by PFS, radiotherapy was superior to other therapies for the whole cohort (*p*=0.029) and recurrence only within the pelvic cavity (*p*=0.005), but the advantages of radiotherapy disappeared in patients with a history of radiotherapy (*p* values >0.05). For recurrence only beyond the pelvic cavity, combination therapy resulted in improved PFS (*p*=0.028). For recurrence both within and beyond the pelvic cavity, no therapy regimen provided additional PFS benefits (*p* values >0.05). Radiotherapy and combination therapy were also associated with improved postrecurrence OS for recurrence within the pelvic cavity (*p*=0.034) and only beyond the pelvic cavity (*p*=0.017), respectively.

**Conclusions:**

In cervical cancer patients, postrecurrence radiotherapy can improve PFS and OS for patients with recurrence within the pelvic cavity and without prior radiotherapy. For recurrence beyond the pelvic cavity or cases with a history of radiotherapy, combination or individualized therapy may provide potential survival benefits.

## Introduction

Uterine cervical cancer is one of the most common causes of female cancer-related death among women worldwide (1). The recurrence rates of cervical cancer are 11% to 22% and 28% to 64% for those with Federation of Gynecology and Obstetrics (FIGO) stage IB-IIA and IIB-IVA disease, respectively (2). Some studies have reported that the recurrence rate for those with stage III to IVB is as high as 70% (3, 4). The treatment for recurrent cervical cancer remains challenging, and the prognosis of recurrent cervical cancer remains poor, with a 5-year overall survival (OS) rate less than 5% despite intensive therapy (5–8) particularly for those who experience a recurrence in a previously irradiated field (9–11). The poor prognosis of recurrent cervical cancer is attributed to several factors, including the biological behavior of the tumor, contraindication of repeated radiotherapy for the same field, limited response to systemic chemotherapy or targeted therapy (12, 13) and the uncertain role, indication, and extent of surgical therapy. Therefore, treatment selection should consider the critical factors potentially influencing the prognosis and the advantages/limitations of the therapy itself. However, due to the complex characteristics of recurrent cervical cancer and the scarcity of reliable evidence, treatment selection is challenging and highly individualized, and current guidelines provide only general principles (14, 15).

In this study, based on a cohort of recurrent cervical cancer patients from a tertiary hospital, we aimed to determine the impact of various treatments on progression-free survival (PFS) and OS after recurrence. The clinicopathological characteristics of patients were considered calibration factors in determining the prognostic significance of treatment regimens in the study population. Subgroups were categorized according to the recurrence site and prior radiotherapy history.

## Methods

### Ethical Approval

The institutional review board from the study center approved the study (No. ZS-1427). All patients or their caregivers provided written informed consent before accepting treatment for cervical cancer. All procedures in the study involving human participants were performed in accordance with the ethical standards of the institutional and National Research Committee and with the 1964 *Declaration of Helsinki* and its later amendments or comparable ethical standards.

### Study Design

This was a retrospective pilot study in a tertiary teaching hospital. Detailed epidemiological, treatment and follow-up data were collected from case reports between March 2012 and April 2018. The follow-up ended on March 1, 2020. The primary objective was to investigate the impact of treatment regimens on PFS and OS after first recurrence or progression.

### Patient Enrollment

All patients undergoing intent-to-cure treatment for recurrent uterine cervical cancer at the study center during the study period were included for deliberate review. The inclusion criteria consisted of the following: (1) histological pathology of squamous cell carcinoma, endocervical adenocarcinoma, or adenosquamous carcinoma; (2) acceptance of primary treatment and customized follow-up in the study center; and, (3) acceptance of treatment for recurrent lesions in the study center. Patients were excluded if they did not meet the inclusion criteria, were lost to follow-up after primary treatment, or had no definite clinicopathological information.

### Interventions

#### Evaluation of Primary Tumors and Treatment

Data on the clinicopathological features of the patients and primary tumors were collected. The histological subtypes and differentiation levels were reviewed and confirmed by two independent pathologists (HW and YY). In this study, early stages, locally advanced stages, and advanced stages were defined as stage IA1 to IB1, IB2 to IIB, and III to IVB, respectively. If a patient accepted an intent-to-cure treatment protocol (such as radical radiotherapy/concurrent chemoradiotherapy [CCRT] or radical hysterectomy), the treatment was defined as primary treatment. Adjuvant therapy consisted of entities used before and/or after the primary treatment, including chemotherapy, radiotherapy, or CCRT. According to primary treatment regimens, patients were divided into subgroups of only radiotherapy or CCRT, radiotherapy or CCRT plus chemotherapy, and surgery with/without adjuvant therapy.

#### Diagnosis of Recurrence

This study confirmed recurrence by pathological review and/or imaging evaluations. The recurrence sites were further categorized as within and/or beyond the pelvic cavity. Based on surgical and/or imaging findings, the number of recurrent lesions was categorized as solitary (continuous nodules or masse) or multiple (separated lesions). The diagnostic methods used in this cohort are described elsewhere (16). In general, every 12 months, imaging assessment, including computed tomography (CT), magnetic resonance imaging (MRI), or positron emission tomography-computed tomography (PET-CT) was performed according to the preference of the patient and the potential necessity for disease evaluation.

#### Postrecurrence Treatment and Follow-Up

Postrecurrence treatments consisted of surgery, radiotherapy, chemotherapy, ablation, or their combinations (at least two types of treatment entities). For combination therapy, patients underwent each therapy modality within four weeks after the last date of the previous modality. For patients who underwent surgical treatment, radiotherapy and/or chemotherapy was administered as adjuvant therapy within four weeks after surgery. For patients who did not undergo surgical treatment, chemotherapy was administered as adjuvant therapy or as concurrent therapy along with radiotherapy within four weeks after the last treatment. Anti-angiogenic therapy (bevacizumab or apatinib) and immunotherapy (monoclonal antibody against programmed cell death protein 1 [anti-PD-1]) were administered to some patients. The surgical treatment consisted of resection of lesions and pelvic exenteration. Chemotherapy regimens were recorded as various lines. The follow-up protocols were the same as those used after primary treatment in patients with recurrence who achieved complete remission (CR) or disease-free status.

As there is no consensus on the follow-up of patients after recurrence, the follow-up protocol used for this cohort followed the protocol for primary disease, which has been described previously. However, the recurrence sites were given more attention during the follow-up period through extensive, individualized evaluation of symptoms, serum biomarkers, and imaging evaluation.

### Measures

According to the Response Evaluation Criteria in Solid Tumors (RECIST) version 1.1, (17), clinical remission was confirmed by imaging evaluation, which was performed at least six weeks after the last treatment, or was confirmed by physical examination with or without a biopsy. For patients accepting chemotherapy, if one regimen was initiated within six weeks after the last regimen, it was defined as a secondary line.

### Statistical Analysis

A comparison of continuous variables with a normal distribution was conducted using parametric methods. Nonnormally distributed continuous variables, and categorical data were compared using nonparametric tests. Cox proportional hazard regression analysis was used to investigate the effectiveness of various treatment regimens on PFS and OS. This regression model incorporated factors of histological pathology, primary stage category, primary treatment regimen, and treatment regimen for recurrence. Specifically, a comparison was made between patients with and without primary surgical treatment. Unless otherwise stated, all analyses were performed with a two-sided significance level of 0.05 using Statistical Product and Service Solutions (SPSS) Statistics 21.0 software (IBM Corporation, Armonk, NY, USA).

## Results

### Clinicopathological Characteristics of the Patients

**Figure 1:**
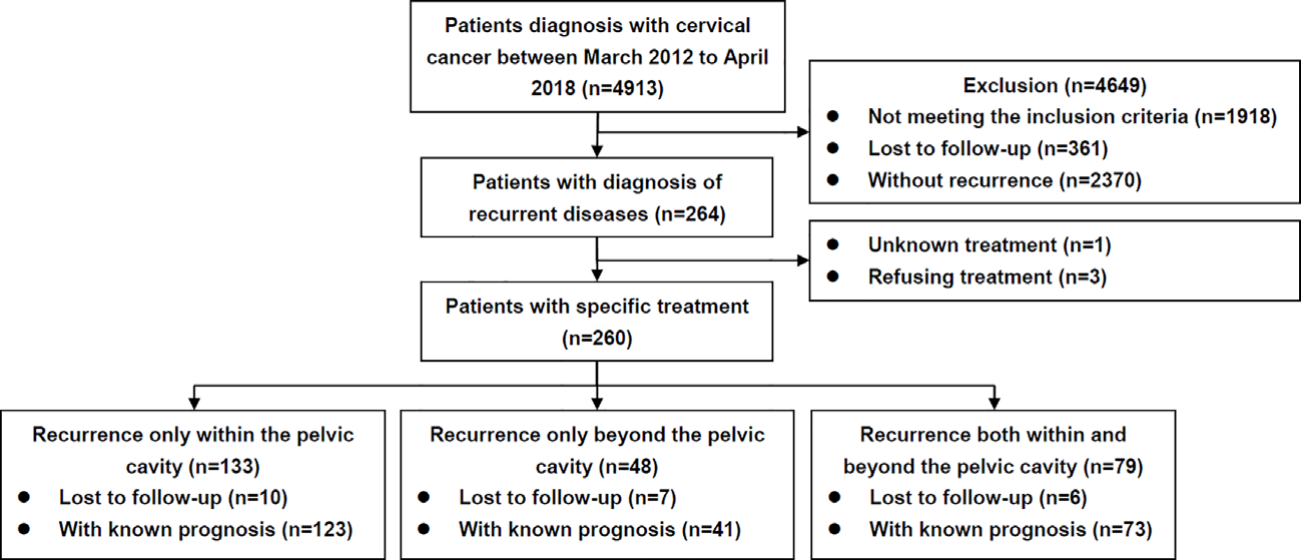
Flow diagram of the study.

A flow diagram is presented in **Figure 1**. The clinicopathological characteristics of the patients are listed in **Table 1**. During the study period, 260 patients diagnosed with and treated for recurrent disease were identified. Overall, 133 (51.2%), 48 (18.5%), and 79 (30.4%) cases experienced recurrence within, beyond, and both within and beyond the pelvic cavity, respectively.

**Table 1:** Demographic and clinicopathological characteristics of the patients who experienced recurrence.

	All patients (n=260)
Age (years), mean ± SD	48.6 ± 9.7
FIGO 2009 staging, n (%)	
I	141 (54.2)
II	101 (38.8)
III	13 (5.0)
IV	5 (1.9)
Staging categories, n (%)	
Early (FIGO stage IA1 to IB1)	103 (39.6)
Locally advanced (FIGO stage IB2 to IIB)	139 (53.5)
Advanced (FIGO stage III to IVB)	18 (6.9)
Histological subtypes, n (%)	
SCC	206 (79.2)
ADC	45 (17.3)
Adenosquamous carcinoma	9 (3.5)
Histological differentiation, n (%)	
Grade 1	19 (12.0)
Grade 2	78 (49.4)
Grade 3	61 (38.6)
Primary treatment regimens, n (%)	
Only chemotherapy	1 (0.4)
Only radiotherapy or CCRT	73 (28.1)
Radiotherapy or CCRT plus chemotherapy	13 (5.0)
Surgery with/without adjuvant therapy	173 (66.5)
Primary treatment with/without radiotherapy, n (%)	
With radiotherapy or CCRT	155 (59.6)
Without radiotherapy or CCRT	105 (40.4)
DFS after first treatment (months), median (range)	17.0 (3.1-249.6)
Recurrent sites, n (%)	
Only within pelvic cavity	133 (51.2)
Only beyond pelvic cavity	48 (18.5)
Both within and beyond pelvic cavity	79 (30.4)
Number of recurrent sites, n (%)	
Solitary	57 (21.9)
Multiply	203 (78.1)
Diagnostic regimens, n (%)	
Symptoms	114 (43.8)
Physical examination	33 (12.7)
Cervical cytology with/without hrHPV	4 (1.5)
Serum biomarker	34 (13.1)
Imaging	75 (28.8)
Pathological evidences of recurrence, n (%)	
No	100 (38.5)
Yes	160 (61.5)
Treatment for recurrence, n (%)	
Radiotherapy or CCRT	161 (61.9)
Surgery	90 (34.6)
Chemotherapy	137 (52.7)
Ablation therapy	8 (3.1)
Combination therapy, n (%)*	
Yes	109 (41.9)
No	151 (58.1)
Surgical patterns, n (%)	
Resection of lesions	48 (57.8)
Pelvic exenteration	35 (42.2)
Lines of chemotherapy, median (range)	1 (1-5)
Antiangiogenic therapy, n (%)	27 (19.7)

*As about half patients underwent combination therapy, the treatment modality number is not equal to patient number.

ADC, adenocarcinoma; CCRT, concurrent chemoradiotherapy; DFS, disease-free survival; FIGO, International Federation of Gynecology and Obstetrics; hrHPV, high-risk human papillomavirus; SCC, squamous cell carcinoma; SD, standard deviation.

### Treatment Selection for Recurrence

As shown in **Table 1**, 109 (41.9%) and 151 (58.1%) patients underwent combination and single therapy, respectively. The median overall therapy duration was 122 (range 7-308) days. As an individual therapy modality, radiotherapy or CCRT, chemotherapy, surgery, and ablative treatment were utilized in 161 (61.9%), 137 (52.7%), 83 (31.9%), and 7 cases (2.7%), respectively.

For patients receiving combination therapy (n=109, **Supplementary Table 1**), surgery/chemotherapy/radiotherapy, surgery/chemotherapy, surgery/radiotherapy, and radiotherapy/chemotherapy were administered to 16.5% (n=18), 33.9% (n=37), 12.8% (n=14) and 15.4% (n=40) of the patients, respectively.

For patients receiving single therapy (n=151), most accepted radiotherapy (n=90), followed by chemotherapy (n=42), surgery (n=18), and ablation of lesions (n=1). Among patients undergoing surgical treatment (n=83), 48 (57.8%) and 35 (42.2%) cases underwent resection of the lesion and pelvic exenteration, respectively. Twenty-seven patients accepted anti-angiogenic therapy (22 with bevacizumab and 5 with apatinib). Only one patient accepted anti-PD-1 therapy and underwent the therapy after pelvic exenteration.

Among patients with and without a radiotherapy history for primary tumors, differences existed in the utilization of repeated radiotherapy (84/155 [54.2%] vs. 77/105 [73.3%], *p*=0.002) and chemotherapy (96/155 [61.9%] vs. 41/105/[39.0%], *p*<0.001), but not in surgical treatment (53/155 [34.2%], 37/105 [35.2%], *p*=0.862). The site and/or the number of recurrent lesions also had various impacts on the selection of treatment regimens (**Supplementary Table 2**).

### Disease Remission and Survival Outcomes

For 237 patients with definite evaluation of disease remission, 149 cases showed CR or a disease-free status (62.9%), 33 showed PR (13.9%), 14 showed SD (5.9%), and 41 showed disease progression (17.3%). The disease remission rates according to treatment regimen and recurrence site are illustrated in **Supplementary Table 3**. Radiotherapy alone and recurrence within the pelvic cavity had the highest CR/PR rates (70/83 [84.3%] and 103/123 [83.7%], respectively).

The median postrecurrence PFS and OS times were 7.0 (range 0-94) months and 24.0 (1.8-149.1) months, respectively. The 3-year and 5-year postrecurrence PFS rates were 27% and 24%, respectively, and the 3-year and 5-year OS rates were 45% and 39%, respectively. Up to the end of follow-up, most of the patients with disease progression (40/41, 97.6%) died, and the median OS was 11.1 (1.8-32.5) months. The only surviving patients had disease progression after radiotherapy alone and then achieved PR after multiple lines of chemotherapy. For patients with SD/PR/CR (n=196), the cumulative progression rates within 1, 2 and 3 years were 56.1% (n=110), 72.4% (n=142) and 83.7% (n=164), respectively. Up to the end of follow-up, most patients with SD (13/14, 92.9%) had disease progression after a median PFS of 4.4 months (1.5-7.8), and most of them died with a median OS of 17.8 (8.1-34.2) months. For PR and CR patients, the progression rates after treatment were 100.0% (33/33) and 56.4% (84/149), the median PFS times were 4.4 (1.6-7.1) and 16.6 (1.7-94.0) months, and the median OS times were 18.3 (5.1-149.1) and 9.9 (31.4-109.4) months, respectively.

Postrecurrence PFS was significantly associated with recurrence site (**Supplementary Figures 1A, B**) and previous radiotherapy history (**Supplementary 2A**). Postrecurrence OS was also significantly associated with radiotherapy history (**Supplementary 2B**) but not with recurrence site (**Supplementary Figures 1C, D**).

### The Effectiveness of Various Treatment Regimens

**Figure 2:**
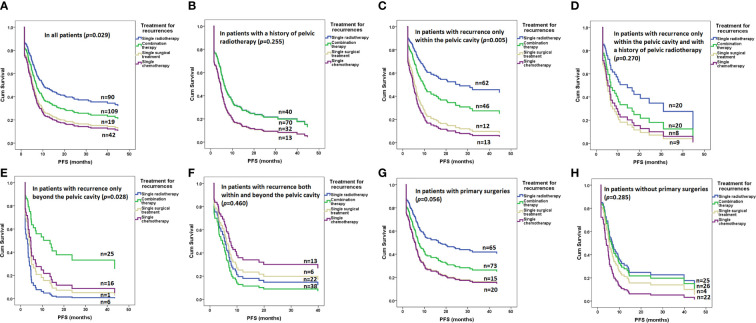
Risk of progression after various treatments for recurrence in all and subgroups of patients determined by a Cox regression model: **(A)** in all patients (*p*=0.029); **(B)** in patients with a history of pelvic radiotherapy (*p*=0.255); **(C)** in patients with recurrence only within the pelvic cavity (*p*=0.005); **(D)** in patients with recurrence only within the pelvic cavity and with a history of pelvic radiotherapy (*p*=0.270); **(E)** in patients with recurrence only beyond the pelvic cavity (*p*=0.028); **(F)** in patients with recurrence both within and beyond the pelvic cavity (*p*=0.460); **(G)** in patients with primary surgeries (*p*=0.056); **(H)** in patients without primary surgeries (*p*=0.285).

As shown in **Figure 2** and **Table 2**, radiotherapy alone resulted in significantly improved PFS compared with other therapies in the whole cohort (*p*=0.029, **Figure 2A**) but not in patients with a radiotherapy history (*p*=0.255, **Figure 2B**). Radiotherapy alone also resulted in significantly improved PFS compared with other therapies in patients with recurrence only within the pelvic cavity (*p*=0.005, **Figure 2C**) but not in patients with a radiotherapy history (*p*=0.270, **Figure 2D**). In patients with recurrence only beyond the pelvic cavity, combination therapy resulted in significantly improved PFS compared with other therapies (*p*=0.028, **Figure 2E**). However, in patients with recurrence both within and beyond the pelvic cavity, none of the therapies resulted in more PFS benefits than other entities (*p*=0.460, **Figure 2F**).

**Table 2:** Independent risk factors for therapeutic effectiveness on progression-free survival determined by Cox regression analysis.

	All patients		Patients with radiotherapy history		Recurrences only within the pelvic cavity		Recurrences only within the pelvic cavity and with radiotherapy history		Recurrences only beyond the pelvic cavity		Recurrences both within and beyond the pelvic cavity	
	HR (95% CI)	*p*	HR (95% CI)	*p*	HR (95% CI)	*p*	HR (95% CI)	*p*	HR (95% CI)	*p*	HR (95% CI)	*p*
Histological pathology		0.271		0.901		0.839		0.561		0.487		0.716
SCC	Reference	–	Reference	–	Reference	–	Reference	–	Reference	–	Reference	–
Endocervical ADC	1.3 (0.8-2.0)	0.202	1.1 (0.6-1.8)	0.830	1.2 (0.6-2.3)	0.594	0.5 (0.2-1.7)	0.282	1.7 (0.3-8.9)	0.503	1.0 (0.5-2.1)	0.901
Adenosquamous carcinoma	1.7 (0.7-3.8)	0.221	1.3 (0.4-4.4)	0.665	1.2 (0.4-4.5)	0.725	0.0 (0.0-N/A)	0.982	2.7 (0.4-17.2)	0.290	1.9 (0.4-8.4)	0.414
Primary stages		0.539		0.258		0.115		0.707		0.097		0.805
Early stages	Reference	–	Reference	–	Reference	–	Reference	–	Reference	–	Reference	–
Locally advanced stages	1.2 (0.8-1.8)	0.271	0.6 (0.4-1.1)	0.102	1.8 (1.0-3.1)	**0.039**	1.5 (0.6-3.8)	0.432	0.5 (0.2-1.7)	0.285	0.9 (0.4-1.7)	0.723
Advanced stages	1.2 (0.6-2.5)	0.685	0.6 (0.3-1.4)	0.242	1.5 (0.3-7.5)	0.643	1.2 (0.2-7.7)	0.855	0.1 (0.02-0.9)	**0.036**	1.2 (0.4-3.9)	0.745
Primary treatment regimens		0.435		0.510		0.891		0.154		0.187		0.098
Only radiotherapy or CCRT	Reference	–	Reference	–	Reference	–	Reference	–	Reference	–	Reference	–
Radiotherapy or CCRT plus chemotherapy	1.2 (0.6-2.6)	0.549	1.5 (0.7-3.2)	0.317	1.0 (0.1-8.7)	0.978	2.1 (0.2-21.7)	0.534	1.3 (0.3-5.8)	0.703	3.8 (1.0-14.1)	**0.045**
Surgery with/without adjuvant therapy	0.8 (0.5-1.2)	0.313	0.9 (0.6-1.5)	0.720	1.2 (0.6-2.2)	0.635	2.1 (1.0-4.6)	0.054	0.4 (0.1-1.1)	0.081	1.0 (0.4-2.0)	0.897
Treatment regimens for recurrence*		**0.029**		0.255		**0.005**		0.270		0.089		0.415
Single radiotherapy	Reference	–	Reference	–	Reference	–	Reference	–	Reference	–	Reference	–
Combination therapy	1.4 (1.0-2.0)	0.088	1.0 (0.6-1.6)	0.974	1.6 (1.0-2.8)	0.072	1.6 (0.7-3.8)	0.264	0.2 (0.1-0.8)	**0.028**	1.2 (0.7-2.4)	0.460
Single Surgical therapy	1.8 (1.0-3.3)	**0.048**	1.6 (0.7-3.3)	0.239	3.0 (1.3-6.7)	**0.009**	2.5 (0.9-6.7)	0.069	0.6 (0.05-7.2)	0.696	0.8 (0.3-2.6)	0.770
Single chemotherapy	2.0 (1.2-3.2)	**0.006**	1.5 (0.9-2.8)	0.142	3.6 (1.6-7.9)	**0.002**	2.2 (0.7-6.6)	0.167	0.5 (0.1-1.9)	0.308	0.6 (0.21.6-)	0.339

*Ablative therapy was combined with surgical therapy.

95% CI, 95% confidential interval; ADC, adenocarcinoma; CCRT, concurrent chemoradiotherapy; HR, hazard ratio; NA, not available. SCC, squamous cell carcinoma.

The bold figures are p values <0.05.

Among 109 patients accepting combination therapy, with radiotherapy/surgery as the reference, chemotherapy/radiotherapy/surgery (HR 3.0, 95% CI 1.1-7.9, *p*=0.028), chemotherapy/surgery (HR 3.3, 1.4-8.0, *p*=0.007) and chemotherapy/radiotherapy (HR 2.6, 1.1-6.3, *p*=0.034) all led to inferior PFS. However, subgroup analysis based on the various recurrence sites provided no significant findings (all *p* values>0.05).

In the Cox regression model, radiotherapy was associated with an improved OS for recurrence within the pelvic cavity (*p*=0.034), even in those with a prior history of radiotherapy (*p*=0.023). Combination therapy also improved OS for recurrence only beyond the pelvic cavity (*p*=0.017). No treatment regimen was associated with an improved OS for recurrence that occurred both within and beyond the pelvic cavity (*p*=0.930). Further details are listed in **Supplementary Table 4**.

### Treatment Selection and Survival Outcomes of Patients Undergoing Primary Surgical Therapy

Generally, 173 (66.5%) patients underwent surgery with or without adjuvant therapy as primary treatment for stage IA1 to IB1 (n=99) or stage IB2 to IIB (n=74) before the first recurrence. The average diagnostic age was 47.2 ± 9.9 years, and the median PFS before first recurrence was 18.3 months (range 3.1-174.5). Open and laparoscopic surgeries were performed on 103 (59.5%) and 70 (40.5%) patients, respectively. Fifty-three patients (30.6%) had metastasis to retroperitoneal lymph nodes, and all underwent chemotherapy. In addition, 68 patients (39.3%) underwent radiotherapy for various risk factors. Only 78 patients (45.1%) received no adjuvant therapy after primary surgery. In univariate analysis, patients with primary surgery had superior PFS (HR 0.7, 95% CI 0.5-0.9, *p*=0.018) and superior OS (HR 0.6, 95% CI 0.4-0.8, *p*=0.001) compared with patients without primary surgery. However, after adjustment for stage and radiotherapy history, patients with primary surgery had a similar PFS (HR 1.0, 95% CI 0.7-1.6, *p*=0.854) and similar OS (HR 0.8, 95% CI 0.5-1.3, *p*=0.352). The risk of progression after various treatments for recurrence in patients with and without primary surgeries is illustrated in **Figures 2G, H**.

## Discussion

Similar to previous findings, the prognosis of recurrent cervical cancer patients was very poor in our study. Treatment of recurrent disease with curative intent requires centralization and involvement of a broad multidisciplinary team (18) and an established network to discuss difficult cases that can refer patients with recurrence for treatment in highly specialized units (19). Our report provides evidence for the selection of treatment protocols according to their effects on postrecurrence survival outcomes. Patients with recurrence limited within the pelvic cavity would benefit from radiotherapy alone, particularly if they have no prior radiotherapy history. For recurrence beyond the pelvic cavity, combination therapy may provide survival advantages. Combination therapy with surgery and radiotherapy seemed to be more effective than other combinations. The results of this study provide a substantial basis that could inform the decision-making process of physicians and patients in terms of treatment, revealing that they should take patient therapy history and the recurrence site into account.

The results of this study, exploring the effectiveness of radiotherapy for local recurrence in cervical cancer patients, reflected those outlined in previous reports (20, 21). Among various combination therapy protocols, radiotherapy plus surgical therapy led to the best prognosis in the whole cohort, reflecting the role of radiotherapy in the treatment of distant metastasis and conditions within the pelvic cavity. These findings suggest that, if possible, incorporating radiotherapy into a combination therapy approach for local or distant recurrence would be helpful.

Where available, platinum-based chemotherapy combined with bevacizumab is the treatment of choice for recurrent disease not amenable to local curative therapy (22). The effectiveness of chemotherapy alone was similar to combination therapy in patients with recurrence both within and beyond the pelvic cavity. In addition, chemotherapy appeared in most combination therapy protocols (83.5%), highlighting its importance in managing distant metastasis and recurrence. However, chemotherapy alone did not demonstrate significant survival benefits for local recurrence in our study. Some authors have even promoted the concept of platinum sensitivity in recurrent cervical cancer (23). However, patients eligible to receive third-line chemotherapy for recurrent cervical cancer can expect minimal benefits at the cost of significant toxicity (24). Other factors, such as the therapy-free interval, may influence the response to chemotherapy and the prognosis of recurrence after definitive CCRT (25).

In our study, surgical therapy alone led to a prognosis that was non-inferior to those associated with other therapies in patients with recurrence only within the pelvic cavity and with a radiotherapy history. Those with recurrence or persistent cervical malignancies would benefit from surgical intervention (26) even in a previously irradiated field (27, 28). For patients with central pelvic relapse without pelvic wall involvement or extrapelvic spread of the disease after previous radiation, pelvic exenteration is usually indicated as the curative approach but has high extensive surgery-related morbidity and mortality (9, 21, 29–31). Pelvic exenteration achieved a 5-year OS of 40% (32, 33) similar to the 5-year OS of 39% in this study. On the other hand, surgical therapy plays an important role in the treatment of recurrence beyond the pelvic cavity, especially for oligometastatic disease (34). It has also been suggested that thermal ablation of recurrent pelvic tumors is technically feasible in selected patients with no treatment alternative (35).

However, no therapy had superior effectiveness to that of others for recurrence both within and beyond the pelvic cavity. These patients had the worst prognosis among the different recurrence site groups. These findings highlight the challenges and difficulties in developing curative therapy for these patients. Novel therapy may break the deadlock in recurrent cervical cancer. Currently, additional effort is needed to develop and test molecular-targeted drugs and immune modulation to achieve improved outcomes for women with recurrent cervical cancer (36).

In our study, patients who underwent surgery for primary disease seemed to have superior survival outcomes. However, after adjustment for disease stage and radiotherapy history, patients who underwent surgery had similar PFS and OS rates. These findings suggest that patients with earlier stages of primary disease had better survival outcomes even after recurrence. However, in multivariate analysis, therapy modality after recurrence had more important effects on survival outcomes.

There were several limitations to our study. Significant heterogeneity exists in this study because it included patients with advanced stages and patients with various primary treatment modalities. In this retrospective cohort, we investigated potential influences from the recurrence site or previous treatment. Still, little is discussed about the patients’ intentions, performance status, adverse effects related to therapy, and available resources. These medical and socioeconomic factors may play equal or more vital roles in the selection and effectiveness of treatment. Anti-angiogenic therapy was utilized in only 27 of 260 (10.4%) patients, which was obviously due to physician preference for treatment and the high cost of the therapy (37) despite its effectiveness in advanced cervical cancer (38). Last, as shown in **Figures 2D–F, H**, in subgroups with small samples of patients, insufficient statistical power produces non-significant results. A larger cohort is needed to illustrate the effects of various treatment protocols further.

## Conclusions

To achieve improved survival outcomes, the selection of therapy for patients with recurrent cervical cancer should consider the previous history of radiotherapy and the site of recurrence because these factors have a significant impact on the effectiveness of treatment. Radiotherapy was the treatment choice in most situations, especially for recurrence limited within the pelvic cavity and in the absence of a history of radiotherapy. For recurrence beyond the pelvic cavity or cases with a radiotherapy history, combination therapy, or individualized therapy may provide potential survival benefits. Combination therapy with surgery and radiotherapy seemed to be more effective than other combinations.

## Data Availability Statement

The datasets presented in this study can be found in online repositories. The names of the repository/repositories and accession number(s) can be found in the article/**Supplementary Material**.

## Ethics Statement

The studies involving human participants were reviewed and approved by The Institutional Review Board of Peking Union Medical College Hospital. The patients/participants provided their written informed consent to participate in this study.

## Author Contributions

LL conceived the original idea for the study, interpreted results, edited the paper, and is the overall guarantor. XC obtained ethical approval, contributed to the preparation of the data set, carried out statistical analysis, interpreted results, and contributed to drafts of the paper. MW, LL, XS, and XC contributed to the study design, interpretation of results, and draft edits. YY and HW conducted the pathological evaluation. All authors contributed to the article and approved the submitted version.

## Funding

This study was supported by the CAMS Innovation Fund for Medical Sciences (CIFMS-2017-I2M-1-002). The funders had no role in the study design, data collection, analysis, decision to publish, or manuscript preparation.

## Conflict of Interest

The authors declare that the research was conducted in the absence of any commercial or financial relationships that could be construed as a potential conflict of interest.
