# Supplementary material for: Selection of Treatment Regimens for Recurrent Cervical Cancer
Source: Front Oncol. 2021 Feb 2;11:618485. doi: 10.3389/fonc.2021.618485 (PMC7884815; doi:10.3389/fonc.2021.618485)

**Supplementary Materials**

[Supplementary Table 1 2](#_Toc53821840)

[Supplementary Table 2 5](#_Toc53821841)

[Supplementary Table 3 6](#_Toc53821842)

[Supplementary Table 4 7](#_Toc53821843)

[Supplementary Figure 1 8](#_Toc53821844)

[Supplementary Figure 2 9](#_Toc53821845)

# Supplementary Table 1

The protocols of combination therapy

| **Num** | **Protocols of combination therapy** |
| --- | --- |
| CC000125 | Chemotherapy/radiotherapy/surgery |
| CC000066 | Chemotherapy/radiotherapy/surgery |
| CC000133 | Chemotherapy/radiotherapy/surgery |
| CC000255 | Chemotherapy/radiotherapy/surgery |
| CC000170 | Chemotherapy/radiotherapy/surgery |
| CC000220 | Chemotherapy/radiotherapy/surgery |
| CC000008 | Chemotherapy/radiotherapy/surgery |
| CC000039 | Chemotherapy/radiotherapy/surgery |
| CC000192 | Chemotherapy/radiotherapy/surgery |
| CC000063 | Chemotherapy/radiotherapy/surgery |
| CC000228 | Chemotherapy/radiotherapy/surgery |
| CC000109 | Chemotherapy/radiotherapy/surgery |
| CC000087 | Chemotherapy/radiotherapy/surgery |
| CC000033 | Chemotherapy/radiotherapy/surgery |
| CC000146 | Chemotherapy/radiotherapy/surgery |
| CC000028 | Chemotherapy/radiotherapy/surgery |
| CC000272 | Chemotherapy/radiotherapy/surgery |
| CC000107 | Chemotherapy/radiotherapy/surgery |
| CC000021 | Chemotherapy/surgery |
| CC000108 | Chemotherapy/surgery |
| CC000128 | Chemotherapy/surgery |
| CC000115 | Chemotherapy/surgery |
| CC000215 | Chemotherapy/surgery |
| CC000131 | Chemotherapy/surgery |
| CC000143 | Chemotherapy/surgery |
| CC000173 | Chemotherapy/surgery |
| CC000264 | Chemotherapy/surgery |
| CC000271 | Chemotherapy/surgery |
| CC000060 | Chemotherapy/surgery |
| CC000097 | Chemotherapy/surgery |
| CC000164 | Chemotherapy/surgery |
| CC000243 | Chemotherapy/surgery |
| CC000095 | Chemotherapy/surgery |
| CC000053 | Chemotherapy/surgery |
| CC000168 | Chemotherapy/surgery |
| CC000056 | Chemotherapy/surgery |
| CC000122 | Chemotherapy/surgery |
| CC000117 | Chemotherapy/surgery |
| CC000061 | Chemotherapy/surgery |
| CC000074 | Chemotherapy/surgery |
| CC000238 | Chemotherapy/surgery |
| CC000166 | Chemotherapy/surgery |
| CC000046 | Chemotherapy/surgery |
| CC000204 | Chemotherapy/surgery |
| CC000139 | Chemotherapy/surgery |
| CC000036 | Chemotherapy/surgery |
| CC000169 | Chemotherapy/surgery |
| CC000230 | Chemotherapy/surgery |
| CC000216 | Chemotherapy/surgery |
| CC000103 | Chemotherapy/surgery |
| CC000098 | Chemotherapy/surgery |
| CC000232 | Chemotherapy/surgery |
| CC000259 | Chemotherapy/surgery |
| CC000144 | Chemotherapy/surgery |
| CC000058 | Chemotherapy/surgery |
| CC000076 | Chemotherapy/radiotherapy |
| CC000096 | Chemotherapy/radiotherapy |
| CC000175 | Chemotherapy/radiotherapy |
| CC000181 | Chemotherapy/radiotherapy |
| CC000043 | Chemotherapy/radiotherapy |
| CC000045 | Chemotherapy/radiotherapy |
| CC000090 | Chemotherapy/radiotherapy |
| CC000111 | Chemotherapy/radiotherapy |
| CC000120 | Chemotherapy/radiotherapy |
| CC000176 | Chemotherapy/radiotherapy |
| CC000185 | Chemotherapy/radiotherapy |
| CC000199 | Chemotherapy/radiotherapy |
| CC000205 | Chemotherapy/radiotherapy |
| CC000269 | Chemotherapy/radiotherapy |
| CC000178 | Chemotherapy/radiotherapy |
| CC000113 | Chemotherapy/radiotherapy |
| CC000177 | Chemotherapy/radiotherapy |
| CC000023 | Chemotherapy/radiotherapy |
| CC000188 | Chemotherapy/radiotherapy |
| CC000156 | Chemotherapy/radiotherapy |
| CC000267 | Chemotherapy/radiotherapy |
| CC000162 | Chemotherapy/radiotherapy |
| CC000182 | Chemotherapy/radiotherapy |
| CC000078 | Chemotherapy/radiotherapy |
| CC000070 | Chemotherapy/radiotherapy |
| CC000069 | Chemotherapy/radiotherapy |
| CC000226 | Chemotherapy/radiotherapy |
| CC000206 | Chemotherapy/radiotherapy |
| CC000187 | Chemotherapy/radiotherapy |
| CC000256 | Chemotherapy/radiotherapy |
| CC000211 | Chemotherapy/radiotherapy |
| CC000084 | Chemotherapy/radiotherapy |
| CC000154 | Chemotherapy/radiotherapy |
| CC000124 | Chemotherapy/radiotherapy |
| CC000174 | Chemotherapy/radiotherapy |
| CC000247 | Chemotherapy/radiotherapy |
| CC000180 | Chemotherapy/radiotherapy |
| CC000044 | Chemotherapy/radiotherapy |
| CC000017 | Chemotherapy/radiotherapy |
| CC000092 | Chemotherapy/radiotherapy |
| CC000031 | Radiotherapy/surgery |
| CC000126 | Radiotherapy/surgery |
| CC000240 | Radiotherapy/surgery |
| CC000059 | Radiotherapy/surgery |
| CC000048 | Radiotherapy/surgery |
| CC000149 | Radiotherapy/surgery |
| CC000147 | Radiotherapy/surgery |
| CC000005 | Radiotherapy/surgery |
| CC000007 | Radiotherapy/surgery |
| CC000160 | Radiotherapy/surgery |
| CC000018 | Radiotherapy/surgery |
| CC000012 | Radiotherapy/surgery |
| CC000050 | Radiotherapy/surgery |
| CC000194 | Radiotherapy/surgery |

# Supplementary Table 2

The association of recurrent sites and numbers with treatment regimens.

|  | Recurrent sites | | | | Recurrent number | | |
| --- | --- | --- | --- | --- | --- | --- | --- |
|  | Only within the pelvic cavity (n=133) | Only beyond the pelvic cavity (n=48) | Both within and beyond the pelvic cavity (n=79) | *p* | Solitary (n=57) | Multiple (n=203) | *p* |
| Combination therapy, n (%) | 46 (34.6%) | 25 (52.1%) | 38 (48.1%) | **0.045** | 27 (47.4%) | 82 (40.4%) | 0.346 |
| Radiotherapy, n (%) | 92 (69.2%) | 24 (50.0%) | 45 (57.0%) | **0.035** | 43 (75.4%) | 118 (58.1%) | **0.017** |
| Surgical therapy, n (%) | 45 (33.8%) | 11 (22.9%) | 27 (34.2%) | 0.333 | 20 (35.1%) | 63 (31.0%) | 0.562 |
| Chemotherapy, n (%) | 50 (37.6%) | 39 (81.3%) | 48 (60.8%) | **<0.001** | 24 (42.1%) | 113 (55.7%) | 0.070 |
| Anti-angiogenic therapy, n (%) | 10 (7.5%) | 4 (8.3%) | 13 (16.5%) | 0.104 | 5 (8.8%) | 22 (10.8%) | 0.651 |

# Supplementary Table 3

The diseases remission rates according to treatment regimens and recurrent sites.

|  | Disease remission, n (%) | | | | | Combined disease remission categories, n (%) | | |
| --- | --- | --- | --- | --- | --- | --- | --- | --- |
|  | Progression | SD | PR | CR | *p* | Progression/SD | PR/CR | *p* |
| Treatment regimens |  |  |  |  | <0.001 |  |  | <0.001 |
| Sole radiotherapy (n=83) | 8 (9.6) | 5 (6.0) | 15 (18.1) | 55 (66.3) |  | 13 (15.7) | 70 (84.3) |  |
| Sole chemotherapy (n=33) | 11 (33.3) | 5 (15.2) | 8 (24.2) | 9 (27.3) |  | 16 (48.5) | 17 (51.5) |  |
| Sole surgery (n=18) | 7 (38.9) | 0 (0.0) | 0 (0.0) | 11 (61.1) |  | 7 (38.9) | 11 (61.1) |  |
| Combination therapy (n=103) | 15 (14.6) | 4 (3.9) | 10 (9.7) | 74 (71.8) |  | 19 (18.4) | 84 (81.6) |  |
| Recurrent sites |  |  |  |  | 0.059 |  |  | 0.030 |
| Only within the pelvic cavity | 18 (14.6) | 2 (1.6) | 17 (13.8) | 86 (69.9) |  | 20 (16.3) | 103 (83.7) |  |
| Only beyond the pelvic cavity | 7 (17.1) | 5 (12.2) | 7 (17.1) | 22 (53.7) |  | 12 (29.3) | 29 (70.7) |  |
| Both within and beyond the pelvic cavity | 16 (21.9) | 7 (9.6) | 9 (12.3) | 41 (56.2) |  | 23 (31.5) | 50 (68.5) |  |

# Supplementary Table 4

The independent risk factors for the therapeutic effectiveness of overall survival in Cox regression analysis.

|  | All patients | | Patients with radiotherapy history | | Recurrences only within the pelvic cavity | | Recurrences only within the pelvic cavity and with radiotherapy history | | Recurrences only beyond the pelvic cavity | | Recurrences both within and beyond the pelvic cavity | |
| --- | --- | --- | --- | --- | --- | --- | --- | --- | --- | --- | --- | --- |
|  | HR (95% CI) | *p* | HR (95% CI) | *p* | HR (95% CI) | *p* | HR (95% CI) | *p* | HR (95% CI) | *p* | HR (95% CI) | *p* |
| Histological pathology |  | 0.094 |  | 0.067 |  | 0.276 |  | **0.014** |  | 0.166 |  | 0.088 |
| SCC | Reference | - | Reference | - | Reference | - | Reference | - | Reference | - | Reference | - |
| Endocervical ADC | 1.2 (0.7-1.9) | 0.540 | 1.1 (0.6-2.1) | 0.808 | 1.6 (0.8-3.3) | 0.229 | 1.6 (0.6-4.7) | 0.384 | 1.2 (0.1-11.5) | 0.855 | 0.5 (0.2-1.4) | 0.208 |
| Adenosquamous carcinoma | 2.4 (1.1-5.5) | **0.030** | 3.8 (1.2-11.7) | **0.022** | 2.3 (0.7-7.8) | 0.174 | 33.8 (3.0-385.8) | **0.005** | 7.1 (0.9-53.8) | 0.058 | 3.5 (0.8-16.1) | 0.105 |
| Primary stages |  | 0.191 |  | 0.269 |  | 0.064 |  | 0.727 |  | 0.065 |  | 0.852 |
| Early stages | Reference | - | Reference | - | Reference | - | Reference | - | Reference | - | Reference | - |
| Locally advanced stages | 1.4 (0.9-2.2) | 0.120 | 0.7 (1.2-11.7) | 0.260 | 2.1 (1.1-4.2) | **0.025** | 1.5 (0.5-4.9) | 0.498 | 0.8 (0.2-3.0) | 0.782 | 1.1 (0.5-2.4) | 0.859 |
| Advanced stages | 1.0 (0.4-2.3) | 0.912 | 0.4 (0.2-1.2) | 0.109 | 3.5 (0.7-18.3) | 0.136 | 2.1 (0.3-15.6) | 0.458 | 0.05 (0.004-0.7) | **0.029** | 1.5 (0.4-5.7) | 0.583 |
| Primary treatment regimens |  | **0.037** |  | 0.061 |  | 0.464 |  | 0.830 |  | 0.103 |  | 0.172 |
| Only radiotherapy or CCRT | Reference | - | Reference | - | Reference | - | Reference | - | Reference | - | Reference | - |
| Radiotherapy or CCRT plus chemotherapy | 1.5 (0.7-3.3) | 0.320 | 1.6 (0.7-3.9) | 0.247 | 1.3 (0.2-6.6) | 0.769 | 1.3 (0.2-8.0) | 0.750 | 2.0 (0.4-10.7) | 0.402 | 3.6 (0.8-16.4) | 0.104 |
| Surgery with/without adjuvant therapy | 0.6 (0.4-1.0) | **0.035** | 0.6 (0.4-1.0) | 0.079 | 0.7 (0.4-1.4) | 0.279 | 0.8 (0.4-1.9) | 0.673 | 0.3 (0.1-1.0) | 0.060 | 0.8 (0.3-1.8) | 0.521 |
| Treatment regimens for recurrence* |  | 0.058 |  | 0.068 |  | **0.037** |  | **0.023** |  | **0.017** |  | 0.930 |
| Sole radiotherapy | Reference | - | Reference | - | Reference | - | Reference | - | Reference | - | Reference | - |
| Combination therapy | 1.4 (0.9-2.2) | 0.100 | 1.2 (0.7-2.1) | 0.516 | 2.1 (1.1-4.0) | **0.017** | 2.4 (0.9-6.5) | 0.084 | 0.2 (0.05-1.0) | **0.044** | 1.0 (0.5-2.0) | 0.986 |
| Sole Surgical therapy | 2.5 (1.3-4.7) | 0.007 | 2.9 (1.3-6.6) | **0.009** | 3.3 (1.3-8.0) | **0.009** | 5.5 (1.8-16.7) | **0.003** | 11.0 (0.6-190.0) | 0.100 | 1.1 (0.3-3.8) | 0.828 |
| Sole chemotherapy | 1.4 (0.8-2.4) | **0.273** | 1.2 (0.6-2.4) | 0.615 | 2.0 (0.8-5.3) | 0.142 | 1.6 (0.4-6.5) | 0.497 | 0.3 (0.1-1.5) | 0.153 | 0.7 (0.2-2.2) | 0.591 |

* Ablative therapy was combined with surgical therapy.

Abbreviation: 95% CI, 95% confidential interval. ADC, adenocarcinoma. CCRT, concurrent chemoradiotherapy; HR, hazard ratio. NA, not available. SCC, squamous cell carcinoma.

# Supplementary Figure 1

Survival outcomes after recurrence according to recurrent sites in the Kaplan-Meier analysis.

(A) The postrecurrence PFS of patients with different sites of recurrence (*p*=0.001).

(B) The postrecurrence PFS of patients with recurrences within or beyond the pelvic cavity (*p*<0.001).

(C) The postrecurrence OS of patients with different sites of recurrence (*p*=0.068).

(D) The postrecurrence OS of patients with recurrences within or beyond the pelvic cavity (*p*=0.055).

Abbreviation: PFS, progression-free survival. OS, overall survival.


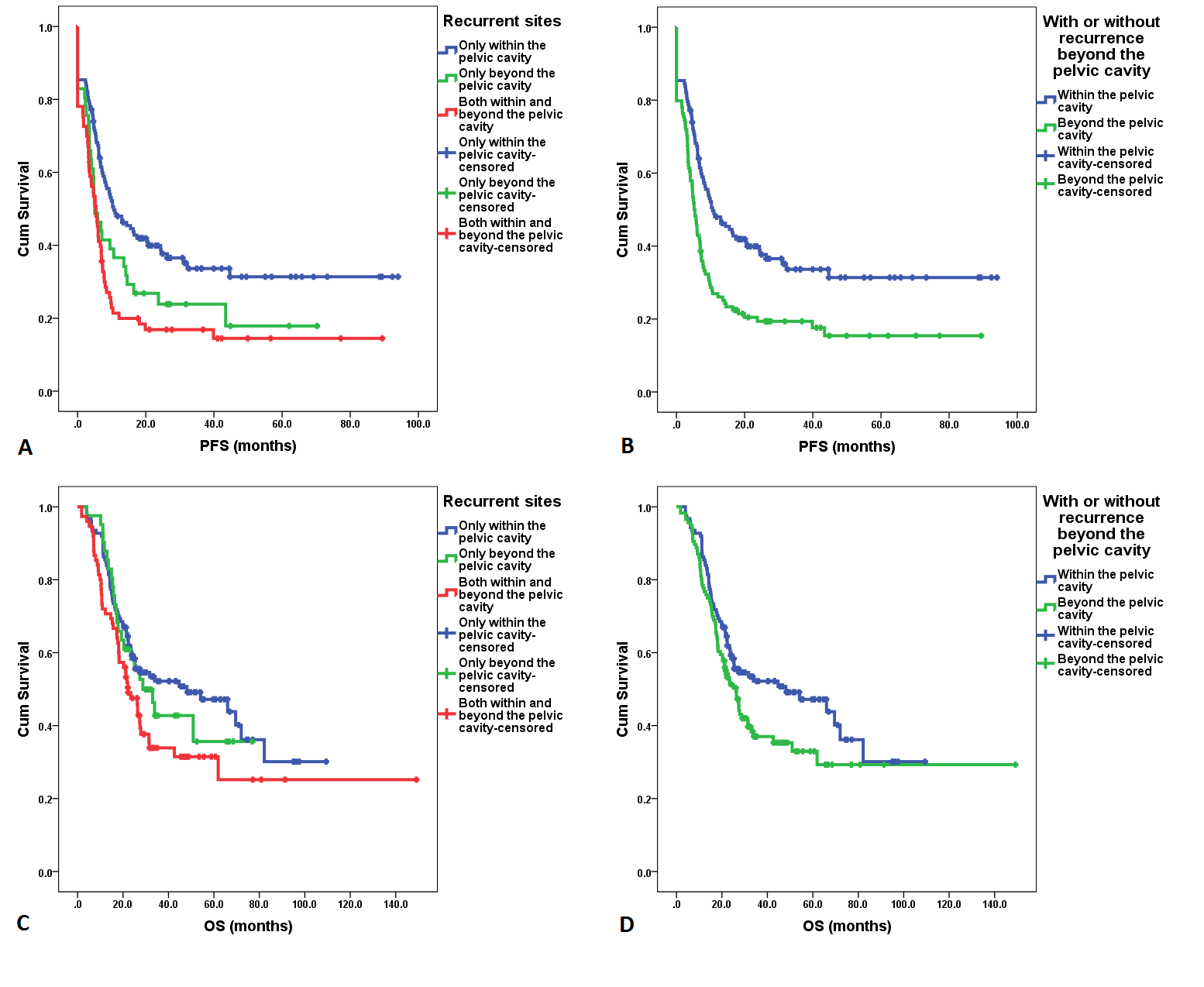


# Supplementary Figure 2

Survival outcomes after recurrence according to radiotherapy history in the Kaplan-Meier analysis.

(A) The postrecurrence PFS of patients with and without radiotherapy history (*p*<0.001).

(B) The postrecurrence OS of patients with and without radiotherapy history (*p*<0.001).

Abbreviation: PFS, progression-free survival. OS, overall survival.


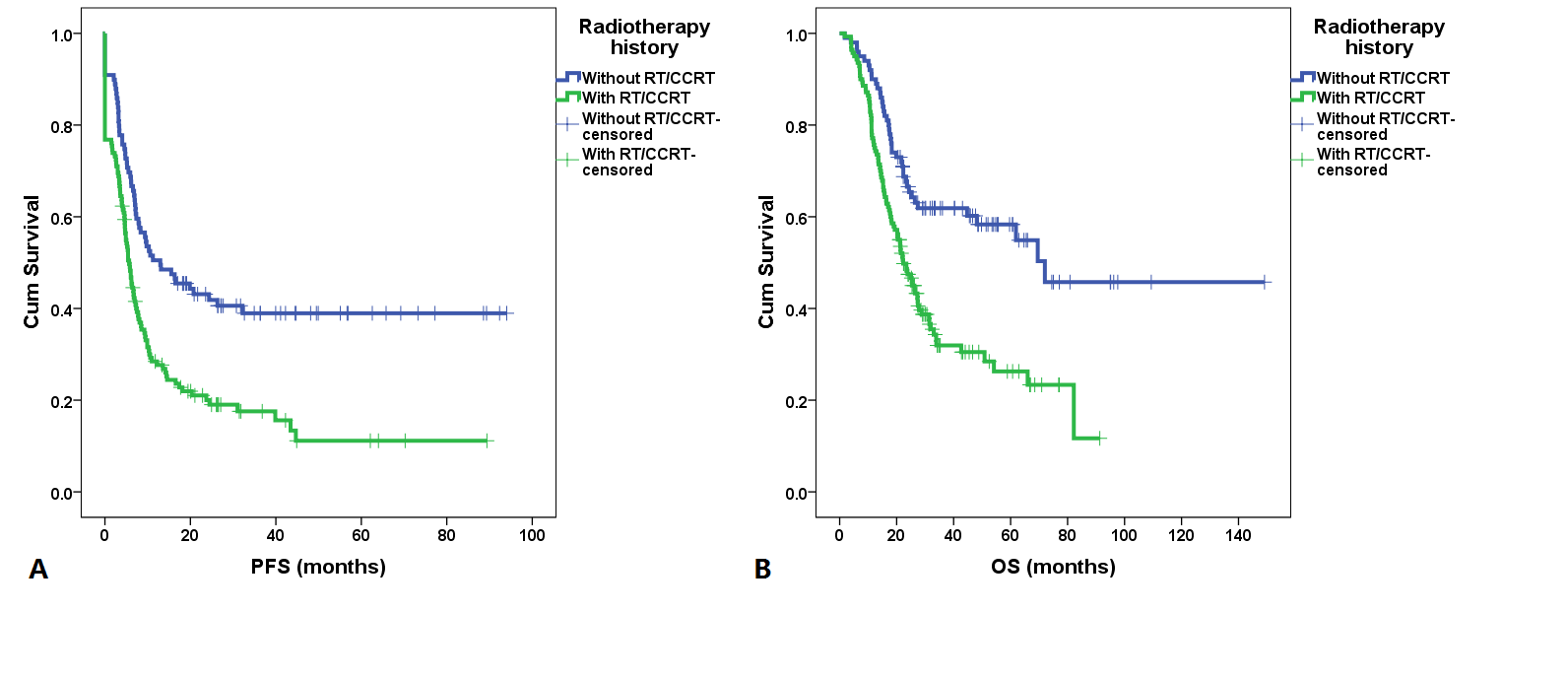

Supplement: Supplementary file 2 [file DataSheet_2.docx]
